# The Clinical Efficacy of Platelet-Rich Plasma versus Conventional Drug Injection in the Treatment of Knee Osteoarthritis: A Study Protocol for a Randomized Controlled Trial

**DOI:** 10.1155/2022/8767137

**Published:** 2022-04-25

**Authors:** Qi Ma, Xiaoyan Hei, Shiqiang Zhu, Xinbao Tian, Yundong Chen, Ning Zhu, Jinchen Zhang, Ping Feng, Yujuan Liu, Lijuan Xing, Xiaojun Li, Qiang Liu, Fengqiao Li, Xiaolong Li, Zheying Lai, Ruizhu Lin, Jianfeng Xu

**Affiliations:** ^1^General Hospital of Ningxia Medical University, Yinchuan, Ningxia, China; ^2^Clinical College of Ningxia Medical University, Yinchuan, Ningxia, China; ^3^People's Hospital of Ningxia Hui Autonomous Region, Yinchuan, Ningxia, China

## Abstract

Knee osteoarthritis is a common chronic degenerative joint disease in middle-aged and elderly people. Intra-articular injection for the treatment of knee osteoarthritis is a regularly utilized nonsurgical treatment in modern medicine. Hyaluronic acid (HA) and platelet-rich plasma (PRP) are two frequently employed intra-articular devices. Hyaluronic acid (HA) is an accepted nonsurgical treatment for symptomatic KOA, and platelet-rich plasma is a popular option in the treatment of KOA in recent years. The purpose of this research is to compare the efficacy and safety of intra-articular injection of platelet-rich plasma (PRP) versus hyaluronic acid (HA) on the pain score scale, knee function, and related inflammatory biomarkers in KOA patients using a clinical randomized controlled trial. Participants are being randomized into either the hyaluronic acid (HA) or into the platelet-rich plasma (PRP) group. All patients receive 4 weeks of treatment (once a week), and well-being support and quadriceps training (3 times a week). The primary outcomes are measured using the Western Ontario and McMaster Universities Osteoarthritis Index (WOMAC) and the visual analog scale (VAS). The secondary outcomes include the activities of daily living score, erythrocyte sedimentation rate, C-reactive protein testing, interleukin-6 levels, and X-ray examination. In order to monitor the occurrence of irregularities and abnormalities, patients are assessed at each visit, and restorative treatment is given if necessary. The results of this clinical trial will verify the efficacy of PRP and HA in the treatment of KOA and provide important evidence for the clinical treatment of KOA. The trial was enlisted at the Chinese Clinical Trial Registry on 26 September 2020 (ChiCTR2000038635).

## 1. Introduction

Knee osteoarthritis (OA) is a chronic, progressive, degenerative joint disease characterized by cartilage degeneration, osteophytes, subchondral sclerosis, and synovitis of the knee [1]. According to the World Health Organization (WHO), knee OA is the fourth most common debilitating disease in the world [2]. The overall prevalence of joint disease in the United States is about 15%, of which knee OA accounts for more than 40% [3]. Relevant statistics show that the rate of knee OA is higher than 50% in individuals over the 60-year-old in China [4]. The essential clinical manifestations of knee osteoarthritis are knee pain, standing difficulties, and severe disability with swelling, deformity, contractures, and muscular atrophy [5]. The disease has a devastating impact on quality of life and imposes a heavy burden on individuals, families, and societies. Therefore, knee osteoarthritis has been officially recognized as a major public health crisis [6].

Current treatment strategies for knee OA include basic health education, oral anti-inflammatory drugs, exercise therapy, physical therapy, topical anti-inflammatory gels, and intra-articular injections [7, 8]. Intra-articular injection is usually the last treatment option before surgical intervention and includes intra-articular corticosteroids or platelet-rich plasma (PRP) or hyaluronic acid (HA).

Hyaluronic acid (HA), the main component of the extracellular matrix, is a polysaccharide composed of glucosamine. Also known as a physiological lubricant for synovial joints, it promotes cell migration and proliferation, possesses anti-inflammatory properties, and regenerates tissue [9, 10]. Experts agree that hyaluronic acid is an effective method for treating knee OA [11], and there is sufficient evidence that hyaluronic acid can effectively relieve pain and improve knee function. Animal experiments have shown that HA plays an important role in anti-inflammatory, antiapoptotic, antiangiogenic, and antifibrosis [12]. A large number of clinical studies have shown that HA injection in patients with KOA can reduce joint pain and improve joint function and quality of life [13].

Platelet-rich plasma (PRP) is an autologous blood product rich in a variety of growth factors. Due to the fact that it contains a large number of growth factors, inflammatory mediating factors, and proteins, it promotes the proliferation of human synovium and chondrocytes to repair damaged cartilage tissue [14, 15]. Current studies demonstrated that intra-articular PRP injection produces greater and longer-lasting improvements in most of the outcome parameters compared to HA [16–18]. Increasing attention is being paid to the use of PRP in the treatment of osteoarthritis. In the past, reports on the efficacy of PRP or HA injection of knee OA mostly focused on the evaluation of clinical efficacy, while there were relatively few basic studies and reports on the changes of the levels of inflammatory cytokines in patients after joint cavity injection.

The onset and progression of osteoarthritis is believed to be associated with inflammation, even in the early stages of the disease. TNF-*α* and IL-6 are important cytokines involved in joint synovitis and cartilage matrix degradation [19]. CRP and ESR levels were positively correlated with OA severity [20]. In this study, patients in the PRP group receive intra-articular injection of PRP combined with quadriceps training; Patients in the HA group receive intra-articular injection of HA combined with quadriceps training. The purpose of this study is to compare the clinical and biological effects of intra-articular injection of PRP versus intra-articular injection of HA in patients with mild to moderate knee OA. The objective of the trial is to determine the clinical viability of PRP within the treatment of knee OA and to provide clinical evidence.

## 2. Methods

### 2.1. Study Design and Setting

**Figure 1:**
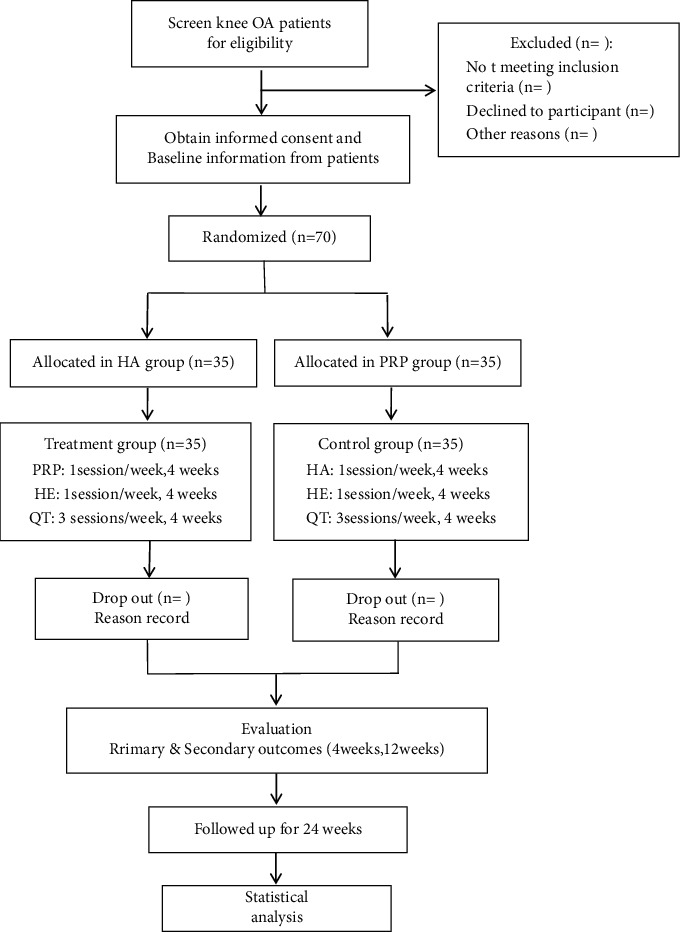
The planned flowchart of the trail. PRP: platelet-rich plasma; HA: hyaluronic acid; HE: health education; QT: quadriceps femoral training; VAS: Visual Analogue Scale; WOMAC: Western Ontario and McMaster University Bone joint Index; ADL: activity of daily living score; ESR: erythrocyte sedimentation rate; CRP: C-reactive protein; IL-6: interleukin-6; X-ray: X-ray examination.

This is a prospective randomized controlled superiority clinical trial to assess the adequacy and security of intra-articular injection of PRP and intra-articular injection of HA for knee osteoarthritis. This overview was conducted at the General Hospital of Ningxia Medical University. A total of 70 participants with knee osteoarthritis are being randomly allocated to the PRP and HA groups at a 1 : 1 ratio. Prior to initiation of the study, all subjects complete daily activity tests after a questionnaire asking for detailed medical history. All study participants are required to sign a written informed consent prior to participation. The study includes assessments at the following time points: before intervention, 1 month after intervention, and after a further follow-up period of 3 and 6 months with no active intervention (Figure 1).

### 2.2. Sample Size Calculation

In this strategy, WOMAC pain score reduction was concluded to be the most relevant indicator based on a previous study conducted by Vaquerizo et al. [21].

The test measure was evaluated using PAS 24.0 criteria, and the total number was set at 70 cases, specifically 35 cases in each group (permitting 20% dropout rate). The power is 80%, and the level of significance is 0.05. The overall sample size is anticipated to be 70 patients (35 in each group) (Table 1).

### 2.3. Participants and Recruitment

A total of 70 participants are being enlisted from the community in Yinchuan, Ningxia Hui Independent Locale, China. Participants are enlisted through multimodal procedures, print notices, WeChat, volunteer proposal, and community exposure. All participants undergo a standard assessment, consisting of a complete sociodemographic and clinical data collection. Prior to this, all participants are asked to read and sign the informed consent.

### 2.4. Inclusion Criteria

Patients are eligible qualify if they fulfill the clinical American College of Rheumatology knee OA criteria. Other inclusion criteria are as follows: (1) aged 40–75 years, (2) graded II-III of Kellgren–Lawrence Radiographic Classification, (3) VAS score of knee joint pain is above 4 out of 10, (4) have received no medication or relevant treatment in the last 3 months, and (5) volunteer to take an interest in the investigation and sign the informed consent.

### 2.5. Exclusion Criteria

Patients who (1) have autoimmune arthropathy; (2) have acute pain of knee OA; (3) have bleeding diathesis or a coagulation disorder; (4) have had a knee joint infection, surgery or radiation therapy within the last three months; (5) have had an injection of glucocorticoid or sodium hyaluronate within the last three months or have had an infection of the skin in an injection area; (6) pregnant and lactating women; (7) pretreatment radiographic KL grade reached IV; (8) suffer from serious organ failure, serious cardiovascular and cerebrovascular diseases, blood diseases or infectious diseases; (9) are participating in other trials that influence the results of this study; and (10) are allergic to eggs or HA proteins were exluded from this study.

### 2.6. Withdrawal Criteria and Management

Participants are allowed to withdraw or are asked to withdraw from the study, if (1) the patient has knee pain that cannot be relieved by intra-articular injection (>8 on the VAS score). In this event, the physician evaluates its severity and terminates the study if necessary. (2) In the event of a serious adverse circumstance such as a severe infection, being in a coma, going into shock, or death, the lead investigator will be contacted imminently and the study will be discontinued with immediate effect. (3) Subjects decide to discontinue treatment at any time and for any reason.

### 2.7. Randomization and Allocation

After meeting the inclusion criteria, 70 qualified participants are being randomly allocated to either the PRP group and the HA group in a 1 : 1 allotment proportion. The irregular grouping sequence is performed concurring to an irregular list of numbers produced by the randomization center of Ningxia Medical University. It is hidden in an opaque sealed envelope.

The random sequence is overseen by particular personnel who have no contact with participants and are not included in the information collection or analysis. All qualified participants receive the following administrations free of charge, assessment, X-ray examination, health education, and quadriceps femoral training (both HA group and PRP group). All participants are strongly advised to follow up all evaluation and treatment plans as much as possible.

### 2.8. Blinding

The grouping of participants is unknown to the result evaluators, information supervisors, and statistical analysts. However, participants are not blinded since each participant is aware of their assigned group after allocation is complete. The evaluation progress is kept secret from the physiotherapists and participants in order to ensure the precision of the test indicators and guarantee the objectivity and reliability of the assessment.

Blinding is maintained throughout the entirety of the research process. After completion of the statistical investigation, the blind code will be unveiled.

### 2.9. PRP Injection Method

The patient is in the supine position with the affected knee flexion: 70°–80° and the feet in the neutral position. For local disinfection, the lower edge of the patella and 1 cm inside and outside of the patellar ligament (internal and external genicus) are selected as puncture points, which are used alternately. A disposable syringe is used to puncture into the joint cavity and 5 ml of the preprepared autogenous PRP is injected. If the joint effusion is present, the effusion is extracted first. After injection, the knee joint undergoes exercise on a passive range of motion and is observed for 20–30 minutes to determine that the subjects have no discomfort. For the first two days, participants are asked to do some light physical activity, limit weight on the affected lower limbs, and use ice packs for 10 minutes every eight hours a day. For mild to moderate pains, a maximum of four 500 mg acetaminophen tablets is permitted, and acetaminophen plus codeine is recommended if/when the pain persisted. With the exception of these painkillers, other drugs are not permitted for up to five days after injection. PRP joint injection is performed once a week for a total of 4 times.

### 2.10. Sodium Hyaluronate Treatment Group

The injection method is the same as that in the PRP test group, and patients are injected with 20 ml of sodium hyaluronate at a time at the joint (produced by Shanghai Baijia One Pharmaceutical Co. Ltd.). Hyaluronic acid is injected once a week, and the clinical efficacy is observed after 4 injections. The researchers give similar guidance and support to both groups.

### 2.11. Quadriceps Muscle Training

All patients begin quadriceps muscle training 24 hours after the intervention, and the training is conducted under the guidance of the therapist. Before each treatment, the preparatory activities, mainly including lower limb active exercise and knee flexor and extension exercise, is conducted for 3–5 minutes. Quadriceps femoral training method: (1) Isometric training: squat training: the patients position the back against the wall with the body straight and feet slightly wider than the shoulders. With toes facing front, the patients slowly squat using both legs for a duration of 2–5 minutes and a total of 5–10 times. Participants must make sure they stand slowly and rest for 5–10 seconds between each squat. (2) Isotonic training: a quadriceps training chair is used for this exercise. The patients take the sitting position and refrain from moving their legs whilst stretching and bending their knee 10 times each for 1 group. There are a total of 6 groups for each training session, with a rest period of 2 minutes between groups. Weight training is also carried out base on standard according to the patient's muscle strength. The weight is gradually increased until the patient can complete the required training volume. The above quadriceps muscle training lasts for 20 minutes each time, once a day, for a total of three times a week. Quadriceps muscle training is ongoing with the whole study process and requires the subjects to keep exercising consistently outside the hospital.

All results are assessed by experienced evaluators who have been trained and specifically allocated to manage these evaluations. Results are randomized blind in accordance after each of the standard visits. Each patient is evaluated by the same evaluator for every stage of the evaluation.

### 2.12. Primary Outcome

1. VAS score [22]: the VAS score is used to assess knee joint pain. It is usually a flat line, 100 mm in length, anchored by word descriptors at each end. Patients mark the point on the line of current pain. The VAS score is then decided by measuring in millimeters from the left end of the line to the point that the patient marked. Patients can experience pain ranging from no pain to extreme pain. The higher the score, the severer the pain. The VAS score is measured during all the assessment visits.
2. Western Ontario and McMaster University Bone joint Index (WOMAC) Scoring System [23]: the WOMAC scale specifically includes 24 questions in three aspects of joint pain, stiffness, and functional limitation. Each question consists of five levels of pain: none, mild, moderate, severe, and very severe. The lowest score for WOMAC is 0, and the highest score is 96 for each item. The higher the score, the worse the joint function.

Variations in VAS scores and WOMAC functional scores for each patient are compared and analyzed at specified time points between groups along with the comparison of the average scores among groups.

### 2.13. Secondary Outcomes

1. Activities of Daily Living (ADL) score [24]: the Improved Balthel Rating Scale is used to measure functional independence (e.g., feeding, dressing, and chair/bed transfer) associated with daily living activities. The score assesses ADL execution and ranges from 0 (completely dependent) to 100 (completely independent).
2. Laboratory indicators: erythrocyte sedimentation rate, C-reactive protein, interleukin-6, and TNF-*α*.
3. Imaging indicators: all patients undergo an X-ray examination (Philips, the Netherlands). During X-ray examination, patients are required to take the positive and lateral radiographs to guarantee reasonable parameters.

### 2.14. Data Management

The study is regularly observed by the Information and Security Observing Board (DSMB) of the clinical assessment center of the General Hospital of Ningxia Medical University. The Data Management and Monitoring Committee is independent of the experimental researchers and does not have any competing interests. The Data Management and Monitoring Committee monitors the overall quality and integrity of the data, reviews the original case report forms, interviews the researchers, verifies adverse event records, and confirms that the study complies with the principles of this protocol.

### 2.15. Statistical Analysis

The SPSS 26.0 software is used for data analysis. All quantitative data are indicated by the percentile calculator. The chi-square test is used if the measurement data are normally distributed, and the data are expressed as mean ± standard deviation. The independent sample *T*-test is utilized to compare the mean values of the two groups. If the measurement information does not adjust to the normal distribution, the interquartile spacing is described and the independent sample Mann-Whitney *U* test is utilized. To assess changes within and between groups, we perform an analysis of variance (ANOVA) on repeated measures. Intentionality analysis and multiple interpolation are used to complete the missing values, and intentionality analysis and conforming protocol analysis are used to determine the sensitivity of the inflammatory outcome efficacy indicators. Statistical significance is assumed *P* less than 0.05.

### 2.16. Safety Assessments

All subjects are asked about any adverse events amid treatment at each visit. All abnormalities reported by the patients, such as persistent joint swelling, increased pain, local reactions such as infection, and other reactions such as allergies, fever, and diarrhea are recorded.

### 2.17. Trial Status

The study is progressing with recruitment and intervention.

## 3. Discussion

Knee osteoarthritis (OA) is a common degenerative chronic disease in which pain and loss of function are the main clinical features leading to treatment [1]. Knee OA is currently lacking in specific therapies. The main objective of this study is to relieve symptoms and improve function, but it still fails to promote the repair of degenerative cartilage or inhibit the progression of the disease [25, 26].

OA is a noninflammatory disease, and the level of inflammation in this group is low, mainly associated with synovitis, which can affect osteoarthritis progression through a proinflammatory mechanism [19].

Preliminary studies have shown that the synovium is an important source of intra-articular cytokines. Local synovial proinflammatory cytokines IL-1*β*, TNF-*α*, and IL-6A are detectable in early osteoarthritis [27] and are related to the disease progression and joint pain of OA [28, 29]. Previous studies have shown that proinflammatory cytokines such as IL-6 and TNF-*α* can promote the generation of cartilage matrix metalloproteinases, thereby mediating articular cartilage destruction [30]. Erythrocyte sedimentation rate (ESR) and C-reactive protein (CRP) are the most common laboratory markers of systemic inflammatory disease [20]. The elevated level of hsCRP and ESR reflected the clinical manifestations of KOA and are positively correlated with the severity of OA [31].

HA is an unbranched polyanionic polymer composed of N-acetyl-D-glucosamine and D-glucosamine, which is the main component of synovial fluid and plays a role in nutrition and protection in the joints [32]. PRP acts as a vector for large growth factors, which have the function of promoting tissue repair and is increasingly being used in the treatment of OA [33]. Regarding the mechanism of action of PRP in the treatment of knee osteoarthritis, a large number of studies have shown that PRP contains highly active growth factors, which can stimulate the proliferation and differentiation of chondrocytes and promote the synthesis of cartilage matrix [34]. At the same time, PRP can inhibit the local inflammation of soft tissues such as synovium to a certain extent, and regulate the local microenvironment of the knee joint [35]. Some reports believe that PRP can inhibit inflammatory factors such as tumor necrosis factor-*α* and interleukin [36] and reduce the inflammatory responses in knee osteoarthritis. In conclusion, PRP can promote tissue repair and regulate inflammation, protect cartilage and promote anabolism, and has a positive effect on re-establishing the dynamic balance of joints [37–39]. While HA only targets at KOA symptoms and plays a lubricating and analgesic role, and it cannot significantly reduce the degradation and destruction of articular cartilage [40]. HA has not been shown to reliably resolve the inflammatory cascades within the joint and can cause an acute response in some patients [13,41].

The safety and efficacy of intra-articular injections of PRP and HA remain controversial. The purpose of this study is to compare the effects of intra-articular injection of plasma PRP versus hyaluronic acid (HA) on pain rating scales, knee joint function, related inflammatory factors and imaging levels in KOA patients through a clinical randomized controlled trial. In one group, PRP is implemented in articular cavity injection in patients with knee OA accompanied with the quadriceps muscle and joint trainings. In another group, patients receive HA injection along with the quadriceps muscle and joint trainings. The two groups of KOA patients receiving intervention are assessed by pain scores, knee joint activity function and related inflammatory biomarkers, as well as the impact of imaging, with the objective that it will provide clinical evidence for PRP treatment of knee OA. The data will be released after the study is finalized.

## Figures and Tables

**Table 1:** Schedule outlining the enrolment, interventions, and assessments for the proposed randomized controlled trial.

Timepoint	Study peroid				
	Enrollment	Intervention period 1-4 (weeks)	Outcome assessment 4 (weeks)	Outcome assessment 12 (weeks)	Outcome assessment 24 (weeks)
Inclusion criteria	×				
Exclusion criteria	×				
Informed consent	×				
Baseline	×				
Randomization and allocation	×				
Intervention		×			
VAS	×		×	×	×
WOMAC	×		×	×	×
ADL	×		×	×	×
ESR	×		×	×	×
CRP	×		×	×	×
IL-6	×		×	×	×
TNF-*α*	×		×	×	×
X-ray	×		×	×	×
Adverse events		×	×	×	×
Reasons of dropout and withdrawals		×	×	×	×

## Data Availability

The authors will share the data after the trial is finished.

## Supplementary Materials

Supplementary Materials SPIRIT 2013 checklist: recommended items to address in a clinical trial protocol and related documents.

## References

[1] Goldman L. H., Tang K., Facchetti L. (2016). Role of thigh muscle cross-sectional area and strength in progression of knee cartilage degeneration over 48 months - data from the Osteoarthritis Initiative. *Osteoarthritis and Cartilage*.

[2] Cross M., Smith E., Hoy D. (2014). The global burden of hip and knee osteoarthritis: estimates from the Global Burden of Disease 2010 study. *Annals of the Rheumatic Diseases*.

[3] Deshpande B. R., Katz J. N., Solomon D. H. (2016). Number of persons with symptomatic knee osteoarthritis in the US: impact of race and ethnicity, age, sex, and obesity. *Arthritis Care & Research*.

[4] Bijlsma J. W., Berenbaum F., Lafeber F. P. (2011). Osteoarthritis: an update with relevance for clinical practice. *The Lancet*.

[5] Youm J., Chan V., Belkora J., Bozic K. J. (2015). Impact of socioeconomic factors on informed decision making and treatment choice in patients with hip and knee OA. *The Journal of Arthroplasty*.

[6] Brand C., Buchbinder R., Luka A. (2009). *Guideline for the Non-surgical Management of Hip and Knee Osteoarthritis RAustCollGenPract2009DU*.

[7] Mcalindon T. E., Bannuru R. R., Sullivan M. C. (2014). OARSI guidelines for the non-surgical management of knee osteoarthritis. *Osteoarthritis and Cartilage*.

[8] Hawker G. A., Mian S., Bednis K., Stanaitis I. (2011). Osteoarthritis year 2010 in review: non-pharmacologic therapy. *Osteoarthritis and Cartilage*.

[9] Litwiniuk M., Krejner A., Speyrer M. S., Gauto A. R, Grzela T (2016). Hyaluronic acid in inflammation and tissue regeneration. *Wounds: A Compendium of Clinical Research and Practice*.

[10] Solis M. A., Chen Y. H., Wong T. Y., Bittencourt V. Z, Lin Y. C, Huang L. L (2012). Hyaluronan regulates cell behavior: a potential niche matrix for stem cells. *Biochemistry research international*.

[11] Bannuru R. R., Osani M. C., Vaysbrot E. E. (2019). OARSI guidelines for the non-surgical management of knee, hip, and polyarticular osteoarthritis. *Osteoarthritis and Cartilage*.

[12] Miller L. E., Block J. E. (2013). US-approved intra-articular hyaluronic acid injections are safe and effective in patients with knee osteoarthritis: systematic review and meta-analysis of randomized, saline-controlled trials. *Clinical Medicine Insights. Arthritis and Musculoskeletal Disorders*.

[13] Patel S., Dhillon M. S., Aggarwal S., Marwaha N., Jain A. (2013). Treatment with platelet-rich plasma is more effective than Placebo for knee osteoarthritis. *The American Journal of Sports Medicine*.

[14] Filardo G., Kon E., Roffi A., Di Matteo B., Merli M. L., Marcacci M. (2015). Platelet-rich plasma: why intra-articular? A systematic review of preclinical studies and clinical evidence on PRP for joint degeneration. *Knee Surgery, Sports Traumatology, Arthroscopy*.

[15] Spreafico A., Chellini F., Frediani B. (2010). Biochemical investigation of the effects of human platelet releasates on human articular chondrocytes. *Journal of Cellular Biochemistry*.

[16] Sadabad H. N., Behzadifar M., Behzadifar F., Dehghan H. R (2016). Efficacy of platelet-rich plasma versus hyaluronic acid for treatment of knee osteoarthritis: a systematic review and meta-analysis. *Electronic Physician*.

[17] Cole B. J., Karas V., Hussey K., Merkow D. B., Pilz K., Fortier L. A. (2017). Hyaluronic acid versus platelet-rich plasma: a prospective, Double-blind randomized controlled trial comparing clinical outcomes and effects on intra-articular Biology for the treatment of knee osteoarthritis. *The American Journal of Sports Medicine*.

[18] Saturveithan C., Premganesh G., Fakhrizzaki S. (2016). Intra-articular hyaluronic acid (HA) and platelet rich plasma (PRP) injection versus hyaluronic acid (HA) injection alone in patients with Grade III and IV knee osteoarthritis (OA): a retrospective study on functional Outcome. *Malays Orthop J*.

[19] Kapoor M., Martel P. (2011). Role of Proinflammatory Cytokines in the Pathophysiology of osteoarthritis.. *Nature Reviews Rheumatology*.

[20] Hanada M., Takahashi M., Furuhashi H., Koyama H., Matsuyama Y. (2016). Elevated erythrocyte sedimentation rate and high-sensitivity C-reactive protein in osteoarthritis of the knee: relationship with clinical findings and radiographic severity. *Annals of Clinical Biochemistry: International Journal of Laboratory Medicine*.

[21] Vaquerizo V., Plasencia M. Á., Arribas I. (2013). Comparison of intra-articular injections of plasma rich in growth factors (prgf-endoret) versus Durolane hyaluronic acid in the treatment of patients with symptomatic osteoarthritis: a randomized controlled trial. *Arthroscopy: The Journal of Arthroscopic & Related Surgery*.

[22] Jian Hong Y. E. (2003). Application of Visual Analogue Scale measurement to evaluation of clinical curative effect femoral head necrosis. *China Journal of Modern Medicine*.

[23] Bellamy N., Buchanan W. W., Goldsmith C. H. (1988). Validation study of WOMAC: a health status instrument for measuring clinically important patient relevant outcomes to antirheumatic drug therapy in patients with osteoarthritis of the hip or knee [J]. *Journal of Rheumatology*.

[24] Li Y., Reinhardt J., Gosney J. (2012). Evaluation of functional outcomes of physical rehabilitation and medical complications in spinal cord injury victims of the Sichuan earthquake. *Journal of Rehabilitation Medicine*.

[25] Martino M. M., Briquez P. S., Güç E. (2014). Growth factors engineered for super-affinity to the extracellular matrix enhance tissue healing. *Science*.

[26] Anitua E., Zalduendo M. M., Prado R., Alkhraisat M. H., Orive G. (2015). Morphogen and proinflammatory cytokine release kinetics from PRGF-Endoret fibrin scaffolds: evaluation of the effect of leukocyte inclusion. *Journal of Biomedical Materials Research Part A*.

[27] Jin X., Beguerie J. R., Zhang W. (2013). Circulating C reactive protein in osteoarthritis: a systematic review and meta-analysis. *Annals of the Rheumatic Diseases*.

[28] Stannus O. P., Jones G., Blizzard L., Cicuttini F. M., Ding C. (2013). Associations between serum levels of inflammatory markers and change in knee pain over 5 years in older adults: a prospective cohort study. *Annals of the Rheumatic Diseases*.

[29] Stannus O. P., Jones G., Quinn S. J., Cicuttini F. M., Dore D., Ding C. (2010). The association between leptin, interleukin-6, and hip radiographic osteoarthritis in older people: a cross-sectional study. *Arthritis Research and Therapy*.

[30] Wang-saegusa A., Cugat R., Ares O., Seijas R., Cuscó X., Garcia-Balletbó M. (2011). Infiltration of plasma rich in growth factors for osteoarthritis of the knee short-term effects on function and quality of life. *Archives of Orthopaedic and Trauma Surgery*.

[31] Punzi L., Ramonda R., Oliviero F. (2005). Value of C reactive protein in the assessment of erosive osteoarthritis of the hand. *Annals of the Rheumatic Diseases*.

[32] Reitinger S., Lepperdinger G. (2013). Hyaluronan, a ready choice to fuel regeneration: a mini-review. *Gerontology*.

[33] Hedbom E., Häuselmann H. J. (2002). Molecular aspects of pathogenesis in osteoarthritis: the role of inflammation. *Cellular and Molecular Life Sciences*.

[34] Mifune Y., Matsumoto T., Takayama K. (2013). The effect of platelet-rich plasma on the regenerative therapy of muscle derived stem cells for articular cartilage repair. *Osteoarthritis and Cartilage*.

[35] Textor J. A., Willits N. H., Tablin F. (2013). Synovial fluid growth factor and cytokine concentrations after intra-articular injection of a platelet-rich product in horses. *The Veterinary Journal*.

[36] Sundman E. A., Cole B. J., Karas V. (2014). The anti-inflammatory and matrix restorative mechanisms of platelet-rich plasma in osteoarthritis. *The American Journal of Sports Medicine*.

[37] Tohidnezhad M., Wruck C.-J., Slowik A. (2014). Role of platelet-released growth factors in detoxification of reactive oxygen species in osteoblasts. *Bone*.

[38] Sakata R., McNary S. M., Miyatake K. (2015). Stimulation of the Superficial Zone protein and lubrication in the articular cartilage by human platelet-rich plasma. *The American Journal of Sports Medicine*.

[39] Ude C. C., Sulaiman S. B., Min-Hwei N. (2014). Cartilage regeneration by chondrogenic induced adult stem cells in osteoarthritic sheep model. *PLoS One*.

[40] Vincent H. K., Percival S. S., Conrad B. P. (2013). Hyaluronic acid (HA) viscosupplementation on synovial fluid inflammation in knee osteoarthritis: a pilot study. *The Open Orthopaedics Journal*.

[41] Miller L. E., Block J. E. (2013). US-approved intra-articular hyaluronic acid injections are safe and effective in patients with knee osteoarthritis: systematic review and meta-analysis of randomized, saline-controlled trials. *Clinical medicine Insights, Arthritis and Musculoskeletal Disorders*.

